# Concomitant immunity against superimposed homologous *Echinostoma caproni* infections in mice is mediated by interleuquin-25

**DOI:** 10.1590/0074-02760250004

**Published:** 2025-10-20

**Authors:** Paola Cociancic, Emma Fiallos, José Guillermo Esteban, Carla Muñoz-Antoli, Rafael Toledo

**Affiliations:** 1 Universitat de València, Facultad de Farmacia y Ciencias de la Alimentación, Área de Parasitología, Departamento de Farmacia y Tecnología Farmacéutica y Parasitología, Valencia, Spain

**Keywords:** Echinostoma Caproni, interleuquin-25, Ym1, intestinal helminth, concomitant immunity, resistance

## Abstract

**BACKGROUND:**

The Institute of Cancer Research (ICR) mouse-*Echinostoma caproni* model is used to study mechanisms generating resistance against intestinal helminths due to the development chronic primary infections with Th1 responses, and partial resistance to secondary infections.

**OBJECTIVES:**

This study aimed to evaluate the generation of concomitant immunity against superimposed homologous *E. caproni* infection.

**METHODS:**

Changes in cytokine expression and macrophages markers as a consequence of primary infection, superimposed infection and superimposed infection in anti (α)-interleuquin(IL)-25-treated mice were investigated by real-time polymerase chain reaction (PCR). Translocation and phosphorylation of STAT6 were studied by indirect immunofluorescence (IIF) on intestinal tissue sections. The IIF technique was also used to label M1 and M2 macrophages to confirm the activation pathways.

**FINDINGS:**

Primary *E. caproni* infections elicit partial resistance against homologous superimposed infections. The animal groups displayed distinct patterns in the expression of cytokines, macrophages markers and IL-13Rα2 as well as STAT6 phosphorylation in a process mediated by IL-25. Resistance appears to rely on the ability of to induce IL-13Rα2 upregulation.

**MAIN CONCLUSIONS:**

The concomitant immunity is based the production of IL-25, rather than in the development of Th2 responses. Regarding the IL-25-dependent mechanisms responsible for concomitant immunity, the ability of IL-25 to induce IL-13Rα2 upregulation which serves to limit the production of other regulatory proteins such as Ym1 affecting the maintenance of mucosal homeostasis appears to be critical.

Intestinal helminth infections remain as one of the most prevalent infections worldwide, causing high morbidity.^1^ Intestinal helminth infections provoke chronic and debilitating diseases commonly associated with diarrhoea, abdominal pain, severe anaemia, growth retardation or impaired cognitive function, among other symptoms.^1^ ^,^ ^2^ About 1.5 billion people are affected by intestinal helminth infections, especially in developing countries of Asia, Africa and Latin America, with children being the most affected group by these infections. It was estimated that over 207 million pre-school-age and over 600 million school-age children are infected with one or more species of intestinal helminths.^1^ Moreover, intestinal helminths are the cause of important economic losses in livestock production.^3^ ^,^ ^4^

Despite treatment of intestinal helminth infections is, in general, effective continuous reinfections and emerging resistances, along with the cost of treatment, make its implementation and success difficult. In this context, better understanding of the mechanisms involved in immunity and resistance against intestinal helminths can be of great help for the control of these parasitic diseases.^5^ To this purpose, several host-parasite experimental models have proven to be useful in achieving a better comprehension of the factors determining the resistance to intestinal helminths. Although most of these models have employed nematodes as parasites, studies carried out with trematodes such as members of the genus *Echinostoma* have also been very useful.^6^ ^,^ ^7^

Members of the genus *Echinostoma*, mainly *E. caproni*, have been widely used as experimental models to analyse the factors determining the resistance to intestinal helminths.^6^ ^,^ ^7^ *E. caproni* is an intestinal trematode with no tissue phase in the vertebrate definitive host.^7^ ^,^ ^8^ After infection, the metacercariae excyst in the duodenum and the juvenile worms migrate to the ileum, where they attach to the mucosa. *E. caproni* has a wide range of definitive hosts, although its compatibility differs considerably between rodent species in terms of worm survival and development.^6^ In Institute of Cancer Research (ICR) mice, the infection becomes chronic in relation to the development of a local Th1 response with elevated production of interferon-γ (IFN-γ).^9^ Recent studies of our group showed that partial resistance against *E. caproni* secondary infections is developed after chemotherapeutic cure of a primary infection and innately produced interleuquin(IL)-25 is crucial to determine the resistance. Susceptibility to primary infections was associated with low levels of intestinal IL-25 expression, whilst deworming via administration of praziquantel was accompanied by an increase in IL-25 expression and, in turn, by the onset of a Th2-type response that prevented the establishment of secondary infections.^10^ ^,^ ^11^ However, recent studies have challenged the contribution of IL-25 in both the polarisation of the response towards a Th2 phenotype and the parasite rejection, showing that IL-25, and not Th2 response, is the responsible for resistance against *E. caproni* and also that in secondary *E. caproni* infections in mice, Th2 response is developed independently of the presence of IL-25.^12^ It was suggested that alternative activation of macrophages induced by IL-25 and the activation of mechanisms enhancing the maintenance and healing of mucosal architecture were responsible of resistance to infection.^12^ ^,^ ^13^

Despite concomitant and superimposed infections are the rule in nature, only little attention has been paid to concomitant immunity, especially if compared to the processes of acquired immunity. Probably, the lack of studies on this topic is related to the complexity of this type of interactions and the difficulty entailed in its study.^14^ Herein, we analyse the effect of a primary *E. caproni* in mice on a subsequent homologous superimposed infection. We demonstrate that primary infection induces changes leading to resistance to superimposed infections. The investigation of the mechanisms involved in this feature may serve to achieve a better understanding of the mechanisms involved in resistance to intestinal helminths.

## MATERIALS AND METHODS

*Parasites, hosts and experimental primary and superimposed infections* - The strain of *E. caproni* has been described previously.^15^ Encysted metacercariae of *E. caproni* were removed from the kidney and pericardial cavity of experimentally infected *Biomphalaria glabrata* snails and used to infect male ICR mice by gastric gavage in both primary and superimposed infections (25 metacercariae each) as described before.^16^ Worms collected from primary and superimposed infections were differentiated on the basis of their morphology as previously described.^11^ The positivity of the infection in each case was determined at necropsy or detection of eggs in stools, as described previously.^17^ Animals were maintained under conventional conditions with food and water *ad libitum*.

A total of 40 ICR mice, weighing 30-35 g, were used for the present study. Ten of them were used as negative controls and the remainder 30 animals were randomly allocated into three groups (A, B, and C) of 10 mice each. Animals of the three groups were primarily infected with 25 metacercariae. Mice belonging to the group A, were sacrificed and necropsied at four weeks post-primary infection (wpi). The effect of repeated infection was studied in the animals of the group B. To this purpose, these animals were superinfected with additional 25 metacercariae at two wpi, following the same procedure than in primary experimental infection.

Animals of the group C were used to analyse the effect of IL-25 in the superimposed infections, for which they were sensitised by intraperitoneal injection with specific commercial monoclonal anti-mouse IL-25 (mα-IL-25) (#514418, BioLegend, San Diego, CA, USA). On each of the two days prior to repeated infection, each animal of the group was intraperitoneally injected with mα-IL-25 (concentration 0.25 µg/µL; in 150 µL of saline buffer). Additionally, and to be used as a control of the mα-IL-25-treated mice, five mice were injected with rat IgG1.

**Fig. 1:**
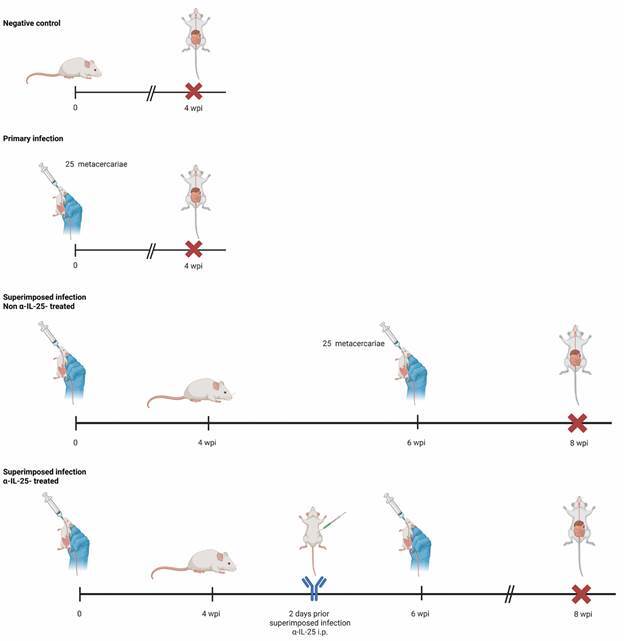
graphical summary of the experimental strategy for the analysis of the factors determining concomitant immunity in superimposed *Echinostoma caproni* infections in mice (wpi: weeks post-infection; α-IL-25: antibodies anti-interleuquin-25; i.p.: intraperitoneal injection).

All the animals with superimposed infections (groups B and C) in the experiment were sacrificed and necropsied at eight wpi. A summary of the experimental design is outlined in Fig. 1.

*Total RNA extraction* - Total RNA was extracted from full-thickness sections of ileum of necropsied mice. Total RNA was isolated using Realclean Tissue/Cells RNA kit (Real Laboratory) according to the manufacturer’s instructions. The cDNA was synthesised using High Capacity cDNA Reverse Transcription kit (Applied Biosystems).

*Real-time polymerase chain reaction (PCR) and relative quantification analysis* - For quantitative PCR, 40 ng total RNA was reverse transcribed to cDNA and added to 10 µL of TaqMan^®^ Universal PCR Master Mix, No AmpErase^®^ UNG (2x), 1 µL of the specified TaqMan^®^ Gene Expression Assay, and water to a final reaction volume of 20 µL. Reactions were performed on the Abi Prism 7000 (Applied Biosystems^®^), with the following thermal cycler conditions: initial setup of 10 min at 95ºC, and 40 cycles of 15 s denaturation at 95ºC and 1 min of annealing/extension at 60ºC each. Samples were amplified in a 96-well plate. In each plate, endogenous control, samples and negative controls were analysed in triplicate. All TaqMan^®^ Gene Expression primers and probes were designed by Applied Biosystems^®^ and offered as Inventoried Assays. The assay ID details are shown in Supplementary data (Table). Each assay contains two unlabelled primers and one 6-FAM™ dye-labelled, TaqMan^®^ MGB probe. Primer concentration was optimised by a matrix of reactions testing a range of concentrations for each primer against different concentrations of the partner primer and also negative controls were included.

Cycle threshold (Ct) value was calculated for each sample, housekeeping and uninfected control. To normalise for differences in efficiency of sample extraction or cDNA synthesis we used β-actin as housekeeping gene. To estimate the influence of infections on the expression levels we used a comparative quantification method (2^-ΔΔCT^ - method). This method is based on the fact that the difference in Ct (ΔCt) between the gene of interest and the housekeeping gene is proportional to the relative expression level of the gene of interest. The fold change in the target gene was normalised to β-actin and standardised to the expression at time 0 (uninfected animals) to generate a relative quantification of the expression levels.

*Indirect immunofluorescence for analysis of STAT6* - Translocation and phosphorylation of STAT6 were studied by indirect immunofluorescence (IIF) on intestinal tissue sections using anti-STAT6 and anti-STAT6P rabbit antibodies (Thermo Fisher Scientific, Waltham, MA, USA).^12^ Paraffin sections were initially dewaxed by incubation at 60ºC for 20 min and hydrated with xylene, graded ethanol (100%, 96% and 70%) and water. Then, an antigen unmasking was performed by boiling in a citrate buffer (10 mM citric acid, 0.05% Tween 20, pH 6.0) to improve antigen detection. Sections were exposed to blocking solution for 1 h at room temperature (RT), washed and then incubated overnight with primary antibody diluted 1/200 (anti-STAT6) or 1/20 (anti-STAT6P) in phosphate-buffered saline (PBS) containing 0.3% Triton X-100-2% bovine serum albumin (BSA) in a humid chamber. After washing, the sections were incubated for 90 min with the secondary antibody, goat anti-rabbit IgG conjugated with Alexa Fluor^®^ 647 (Jackson ImmunoResearch Laboratories, Inc., West Grove, PA, USA) at RT. Finally, the intestinal sections were washed, counterstained with DAPI (1:1000) and mounted using Fluoromount (Sigma-Aldrich, St. Louis, MO, USA). Staining of negative controls without primary antibody was also performed as a negative control of the technique. All incubations were carried out with continuous agitation. Cell staining was observed under a fluorescence microscope and the results were evaluated over 10 high-power fields. ImageJ software was used to measure the mean fluorescence intensities.

STAT6 expression and phosphorylation were quantified using ImageJ software to calculate the percentage of image area covered by Alexa^®^ Fluor 647.^18^ Confocal micrographs (x200) were converted into binary (black and white) images. Raw integrated density (RawIntDen), which is the sum of the values of all pixels in the image, was measured and used to calculate the percentage of area covered by the fluorescent tag (% AC) according to the following formula, in which 255 is the density value of a positive (tagged) pixel in the binary image and areas are expressed in pixels:

$$\%\;AC=\frac{RawIntDen⁄255}{Total\;area}.100$$

*Indirect immunofluorescence for the study of macrophage activation* - The IIF technique was also used to label M1 (classical activation pathway) and M2 (alternative activation pathway) to confirm the macrophage activation pathways.

Ileum sections of the examined mice were dewaxed by incubation at 60ºC for 20 min and hydrated with xylene, graded ethanol (100%, 96% and 70%) and distilled water. Then, samples were kept in a blocking-permeabilising media (5% BSA in PBS-0.2% Triton X-100) for 1 h at RT, followed by a PBS wash. The samples were previously immersed in a citrate buffer (10 mM citric acid, 0.05% Tween 20, pH 6.0). The slides were incubated at 4ºC overnight in a humid chamber with the primary antibody. A rabbit anti-CD16-2/FCGR4/Fc antibody (Sino Biological, clone no. 012), and a goat antibody MR/CD206/Mannose Receptor (Novus Invitro Technologies Ltd, Colorado, USA) were used for labelling M1 and M2, respectively. Both antibodies were diluted in 1% BSA-PBS-0.2% Triton X-100 (1:100 for M1 and 1:200 for M2). A goat anti-rabbit Ig G conjugated with Alexa Fluor^®^ 647 was used as secondary antibody diluted in PBS-0.2% Triton X-100, 1:500 dilutions for M1 and 1:1000 dilutions for M2 during 90 min at RT. Finally, the slides were stained with DAPI and mounted with Fluoromount. Staining of negative controls without primary antibody was also performed as a negative control of the technique. Washes between steps were performed using PBS and the incubations were carried out with continuous agitation. Cell staining was observed under a fluorescence microscope and the results were evaluated over 10 high-power fields.

*Statistical analysis* - To compare the worm recovery between primary and challenge infections, a Student’s *t*-test was used at each week post-infection. One-way analysis of variance (ANOVA) with Bonferroni test as post-hoc analysis were used to compare expression levels of cytokines, enzymes, or other genes analysed by PCR. P < 0.05 was considered as significant. Prior to analyses, data were log transformed to achieve normality and verified by the Anderson-Darling Test.

*Ethical statement* - This study has been approved by the Ethical Committee of Animal Welfare and Experimentation of the University of Valencia and the Generalitat Valenciana (Valencia, Spain) (Ref# 2021/VSC/PEA/0265). Protocols adhered to Spanish (Real Decreto 53/2013) and European (2010/63/UE) regulations.

## RESULTS

*Primary E. caproni infections induce partial resistance against homologous superimposed infections dependent on IL-25* - Our results show that primary *E. caproni* infections elicit partial resistance against homologous superimposed infections. All mice experimentally exposed to 25 metacercariae of *E. caproni* became infected and were positive to egg examination and the subsequent necropsy, both in primary and challenge infections. However, the number of worms collected in primary and superimposed infections was markedly different. In primary infections the number of worms recovered per mouse ranged from 12 to 25 (19.3 ± 4.3) (82.4% of worm recovery) in the animals of the group A. The recovery of worms from primary infection in groups B and C was similar. In contrast, the worm recovery in the superimposed infections varied from 1 to 2 (1.3 ± 0.5) (5.0%). Application of the Student’s *t*-test to the worm recovery in primary and challenge infection showed that the values were significantly lower in superimposed infection than in primary exposures (p < 0.001).

**Fig. 2:**
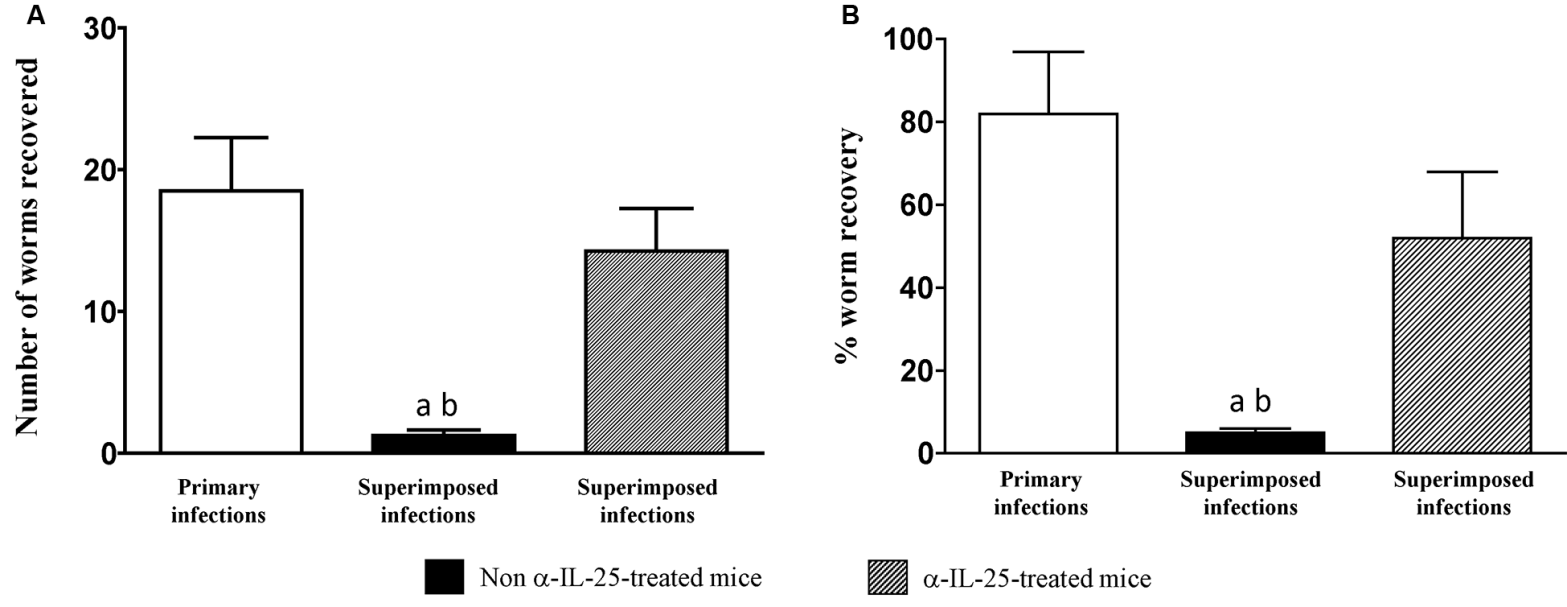
worm recovery significantly decreased in superimposed infections by an interleuquin-25 (IL-25)-dependent mechanism. *Echinostoma caproni* worm recovery was significantly lower in superimposed than in primary infections. Treatment of mice with anti-IL-25 (α-IL-25) antibodies before superimposed infections abrogated the resistance to the challenge infection. (A) Number of worms recovered and (B) percentage of worm recovery in each experimental group. Vertical bars represent the standard deviation. a: significant differences with respect to primary infections; b: significant differences with respect to superimposed infections in α-IL-25-treated mice (p < 0.001).

To analyse the role of IL-25 in the resistance to superimposed infections, a group of primarily infected mice (group C) was treated with anti-IL-25 monoclonal antibodies before the challenge infection. Treatment with anti-IL-25 antibodies partially reverted the resistance to superimposed infections since the number of worms collected from the superimposed infections ranged from 6 to 15 (13.0 ± 16.1) (52.1%), which was significantly higher than in the group of non-treated mice (p < 0.001). Despite this fact, the worm recovery in the animals of the group C, was lower than that observed in primary infections (p < 0.01) (Fig. 2).

**Fig. 3:**
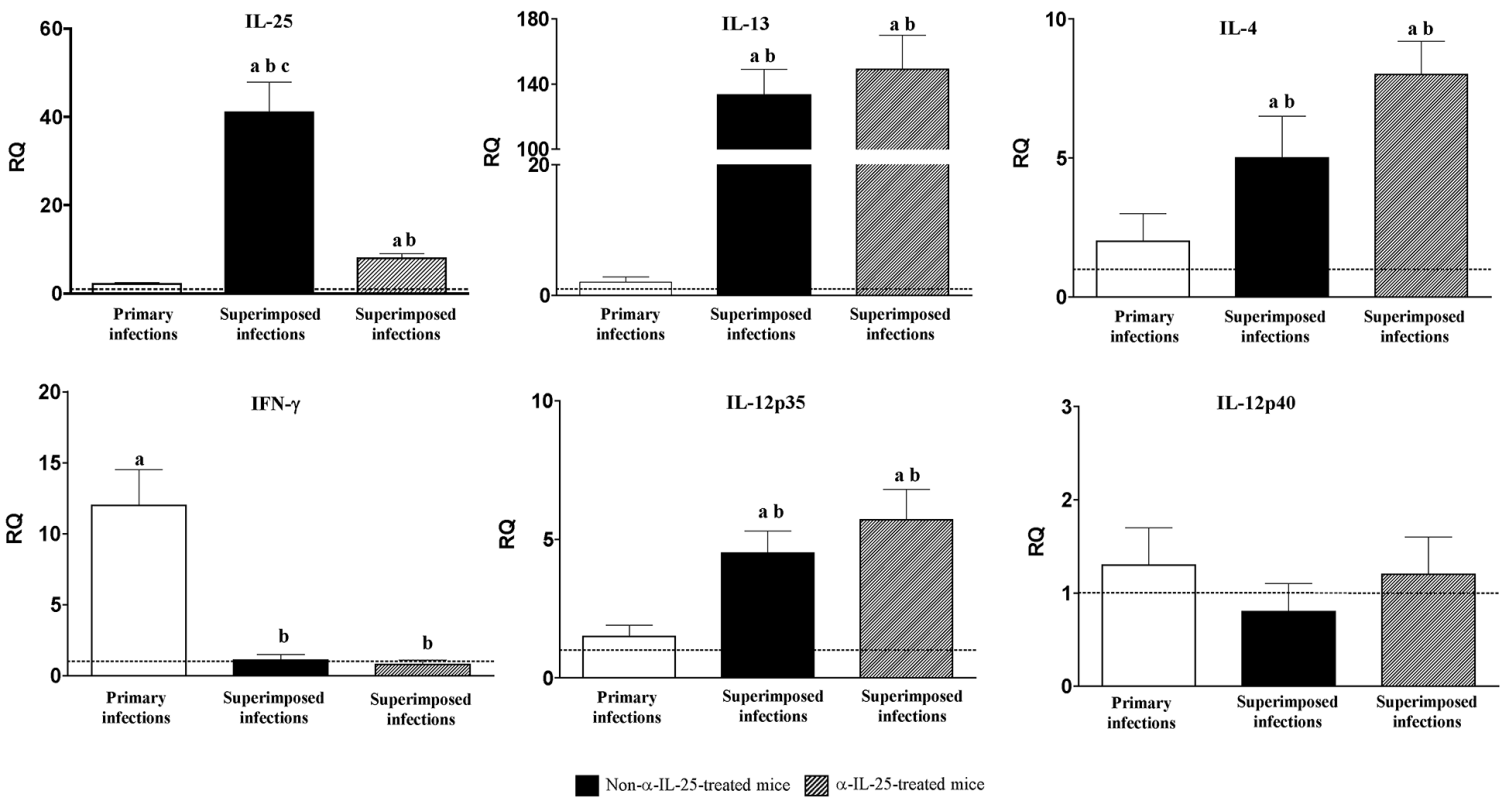
different cytokine profile is associated with primary and superimposed infections. Expression of cytokine mRNA in the intestinal tissue of Institute of Cancer Research (ICR) mice after *Echinostoma caproni* primary, superimposed infection in mice and superimposed infections in anti-interleuquin-25 (α-IL-25)-treated mice. The relative quantities (RQ) of cytokine genes are shown after normalisation with β-actin and standardisation of the relative amount against day 0 sample. Vertical bars represent the standard deviation. a: significant differences with respect to negative controls; b: significant differences with respect to primary infections; c: significant differences with respect to superimposed infection- in α-IL-25-treated mice (p < 0.05).

*Different profile of cytokine expression is associated with primary and superimposed infections* - Changes in cytokine expression as a consequence of primary infection, superimposed infection and superimposed infection in anti (α)-IL-25-treated mice were investigated by real-time PCR and the results are shown in Fig. 3.

The expression of all the cytokines studied, except IL-12p40, became altered over the course of the experiment. Primary infection was characterised by an increase of IFN-γ. In contrast, superimposed infection resulted in a decrease of IFN-γ expression and a marked upregulation of IL-25 and IL-13 and, to a lesser extent of IL-4 and IL-12p35. The pattern of cytokine expression after superimposed in α-IL-25-treated mice was similar to that observed in non-treated mice, except for IL-25. Despite the levels of IL-25 expression were higher than in primary infections, these were significantly lower than in untreated mice (Fig. 3).

**Fig. 4:**
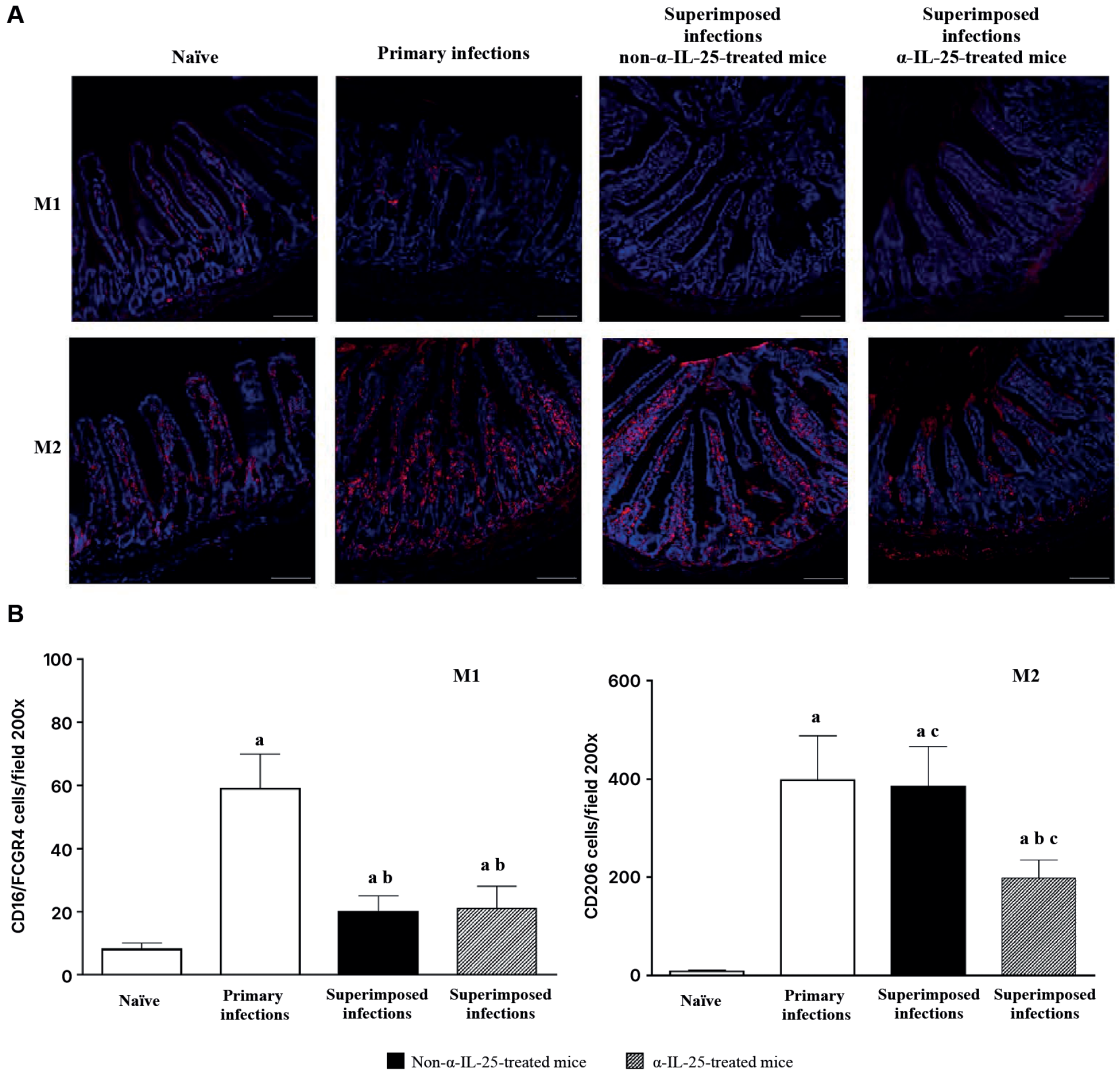
primary and superimposed infection induce different patterns of macrophage activation. (A) Indirect immunofluorescence with recombinant anti-CD16-2/FCGR4/Fc for M1 and antibody MR/CD206/Mannose Receptor for M2 (red) on intestinal tissue of Institute of Cancer Research (ICR) naïve and mice after *Echinostoma caproni* primary, superimposed infection in mice and superimposed infections in anti-interleuquin-25 (α-IL-25)-treated mice (scale bar: 100 mm). (B) Quantitation of the percentage of area covered by anti-CD16-2/FCGR4/Fc for M1 and antibody MR/CD206/Mannose Receptor staining. Vertical bars show standard deviation. a: significant differences with respect to negative controls; b: significant differences with respect to primary infections; c: significant differences with respect to superimposed infection- in α-IL-25-treated mice (p < 0.05).

*Macrophage activation is affected by superimposed infections* - We analysed the macrophage activation in the four experimental groups by indirect immunofluorescence using anti-CD16-2/FCGR4/Fc and MR/CD206/Mannose Receptor antibodies (Fig. 4). Primary infection elicited a marked increase of macrophage activation, especially M2 (Fig. 4A). M1 levels decreased after superimposed infections but, in contrast, M2 levels only decreased after superimposed infection in the α-IL-25-treated group of mice (Fig. 4B).

**Fig. 5:**
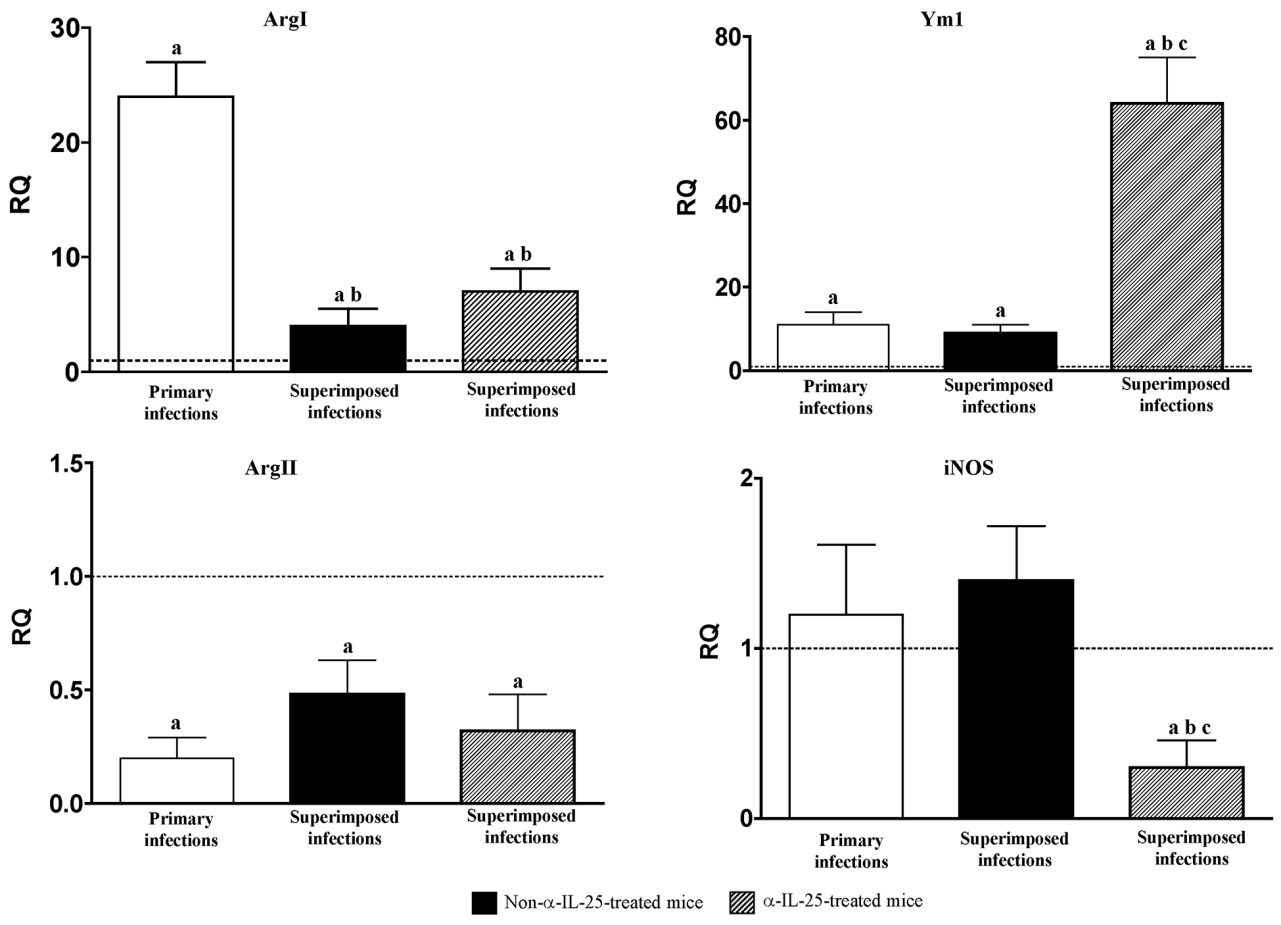
expression of M1 and M2 markers become altered in superimposed infections and anti-interleuquin-25 (α-IL-25) treatment. Pattern of macrophage activation analysed by the expression of markers mRNA of both classical (Arg II and iNOS) and alternative (Arg I and Ym-1) activation of macrophages in the intestinal tissue of Institute of Cancer Research (ICR) mice after *Echinostoma caproni* primary, superimposed infection in mice and superimposed infections in α-IL-25-treated mice. The relative quantities (RQ) of cytokine genes are shown after normalization with β-actin and standardisation of the relative amount against day 0 sample. Vertical bars represent the standard deviation. a: significant differences with respect to negative controls; b: significant differences with respect to primary infections; c: significant differences with respect to superimposed infection- in α-IL-25-treated mice (p < 0.05).

In relation to M1 and M2 markers, no increases were observed in markers of classical activation (ArgII and iNOS) in the entire course of the experiment. Regarding alternative activation markers, primary infection was characterised by a significant upregulation of ArgI and Ym1 (Fig. 5). Despite the level of Ym1 expression remained after superimposed infection, those of ArgI decreased dramatically, although they remained higher than the negative controls and those detected after primary infection. Probably, the most relevant feature is the great increase observed as a consequence of the challenge infection in mice with blocked IL-25 (Fig. 5).

**Fig. 6:**
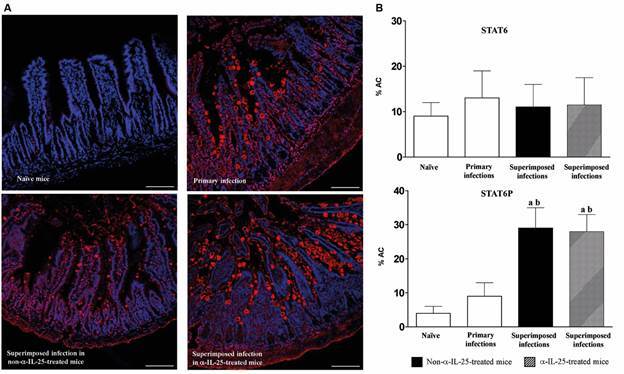
superimposed infection increases STAT6 phosphorylation. (A) Indirect immunofluorescence with anti-signal transducer and activator of transcription 6 (STAT6) and anti-STAT6P (red) on intestinal tissue of Institute of Cancer Research (ICR) naïve and mice after *Echinostoma caproni* primary, superimposed infection in mice and superimposed infections in anti-interleuquin-25 (α-IL-25) treated mice (scale bar: 100 mm). (B) Quantitation of the percentage of area covered by anti-STAT6 and anti-STAT6P staining. Vertical bars show standard deviation. a: significant differences with respect to negative controls; b: significant differences with respect to primary infections (p < 0.05).

*Superimposed infections increase STAT6 phosphorylation* - To evaluate the potential role of STAT6 in the concomitant immunity, we quantified STAT6 and phosphorylated STAT6 by immunofluorescence in the four groups of mice (Fig. 6). The presence of STAT6 was similar in the four experimental groups and it was not affected by the superimposed infections. The percentage of area covered ranged from 9.5 to 14.9 (13.0 ± 5.9) for all the experimental groups of animals. In contrast, a significant increase of STAT6 phosphorylation and translocation was detected as a consequence of the challenge infection. The values of covered area in superimposed infections reached almost four times greater and ranging from 26.9 to 29.7 (28.7 ± 8.0). No significant differences were observed between α-IL-25-treated and non-treated mice after superimposed infections (Fig. 6B).

**Fig. 7:**
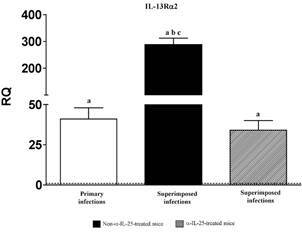
pattern of macrophage activation is different in primary and superimposed infections. Expression of IL-13Ra2 mRNA in the intestinal tissue of Institute of Cancer Research (ICR) mice after *Echinostoma caproni* primary, superimposed infection in mice and superimposed infections in anti-interleuquin-25 (α-IL-25)-treated mice. The relative quantities (RQ) of cytokine genes are shown after normalisation with β-actin and standardisation of the relative amount against day 0 sample. Vertical bars represent the standard deviation. a: significant differences with respect to negative controls; b: significant differences with respect to primary infections; c: significant differences with respect to superimposed infection- in α-IL-25-treated mice (p < 0.05).

*IL-25 regulates the expression of IL-13Ra2* - The expression of the type 2 receptor of IL-13 (IL-13Ra2) was evaluated by real-time PCR (Fig. 7). Our results show that expression of IL-13Ra2 is mediated by IL-25. Primary infection in mice induced upregulation of IL-13Ra2. However, the level of IL-13Ra2 expression was significantly increased as a consequence of the superimposed infection. Strikingly, blocking IL-25 with anti-IL-25 antibodies prior to the superimposed infection returned the expression level to the values of the primary infection (Fig. 7).

## DISCUSSION

Despite mice are susceptible hosts for primary *E. caproni* infections, our results show that infection induces changes in the intestinal environment that result in concomitant immunity against superimposed homologous infections. This immunity is reflected in a lower parasite establishment. Worm recovery decreased from 77.9% in the primary infection to 5.0% in the subsequent superimposed challenge infection. Moreover, our study confirms that resistance to *E. caproni* infections is mediated by IL-25, rather than by the Th2 response as previously described in resistance to secondary *E. caproni* infections.^10^ ^,^ ^12^ The immunological profile observed in concomitant immunity was similar to that detected in resistance to secondary infections. Primary infections elicited a Th1 environment with elevated levels of IFN-γ. In contrast, both types of challenge infection induced a shift toward a Th2 phenotype with upregulation of IL-13 and IL-4 to the detriment of IFN-γ, concomitantly with partial resistance to infection.^9^ ^,^ ^10^ ^,^ ^12^

Resistance to intestinal helminth infections is often associated with the development of local Th2 responses and a complex interplay between innate and adaptive immune cells. IL-4 and/or IL-13 would play a pivotal role since their binding to the IL-4 receptor alpha chain (IL-4Ra) on the surface of several types of cells promotes STAT6 activation and the subsequent activation of effector mechanisms leading to worm rejection.^19^ ^,^ ^20^ ^,^ ^21^ In this context, the attributed role for IL-25 was reduced to act as a driver to the development of the Th2 milieu. However, recent studies have shown that IL-25 is the main responsible for resistance, regardless of the Th2 activity.^10^ ^,^ ^12^ The need of IL-25 against *Nippostrongylus brasiliensis* was demonstrated using IL-25^-/-^, C57BL/6 and G4 IL-4 C57BL/6 reporter mice. No physiological role for IL-25 in either the differentiation of Th2 cells or their development to effector or memory Th2-cell subsets was found for IL-25.^22^ Similarly, mice lacking IL-25 receptors developed normal responses to *Heligmosomoides polygyrus* infection, though the animals were unable to reduce worm burden or parasite egg output.^23^ Our results confirm that the role of IL-25 is not limited to a regulatory function, but it plays a necessary role in resistance to infection. A significant downregulation of IFN-γ, together with an elevation of IL-13, was observed as a consequence of the superimposed infection, concomitantly with resistance to challenge infection. However, blocking of IL-25 using monoclonal anti-IL-25 antibodies abrogated the resistance to superimposed infection without reversion of the Th2 phenotype.

IL-25-related resistance to *E. caproni* infections is due to the generation of a tissue healing environment,^13^ ^,^ ^24^ ^-^ ^29^ though the IL-25-dependent mechanisms for resistance remain unclear. The healing and the regeneration processes of the intestinal mucosa related to the upregulation of IL-25 after the cure of the primary infection were attributed as the main cause for acquired immunity to secondary *E. caproni* infections. IL-25 induced M2 activation and, subsequently, upregulation of ArgI and Ym1. This could modulate the balance between iNOS and ArgI, and the arginase-ornithine decarboxylase metabolic pathway, which constitutes a critical strategy for regulation of the intestinal inflammation and tissue repair.^10^ ^,^ ^12^ ^,^ ^13^ However, our results suggest that mechanisms involved in concomitant immunity are somewhat different from those operating in acquired immunity to secondary infections and depend on an interplay between IL-25, IL-13 and Ym1. Primary *E. caproni* infection elicited an intense macrophage activation that was maintained after superimposed infection, especially M2, as shown by the increased number of CD206+ cells observed in our immunofluorescence analysis. However, the pattern of M2 markers was rather confusing over the course of the experiment. Whereas challenge infection downregulated ArgI both in non-treated and anti-IL-25-treated mice, a strong increase of Ym1 occurred in the treated animals.

Ym1, also known as chitinase-like protein 3 (Chil3), has multifaceted and contradictory regulatory effects. The producing cells of Ym1 are alternatively activated macrophages (M2) and neutrophils, mainly under inflammatory and pathological conditions.^30^ Despite Ym1 has often been used as a marker of M2 differentiation, its function can be opposite depending on the stage of the immune response.^31^ Ym1 has been associated with tissue restorative processes and also with inflammation and exacerbation of tissue pathology by recruitment of neutrophils and upregulation of inflammatory mediators such as IL-17A,^30^ ^,^ ^32^ a common feature in hosts susceptible to *E. caproni*.^16^ ^,^ ^33^ In *N. brasiliensis*-infected mice, Ym1 promoted reparative type 2 responses during the larval migration but, once established an IL-4Rα-dependent response, production of Ym1 was enhanced limiting type 2 responses.^31^ In this context, elevated levels of Ym1 in anti-IL-25-treated mice could impair the intestinal homeostasis promoted by IL-25 and the responsible for concomitant immunity by enhancing parasite survival.

**Fig. 8:**
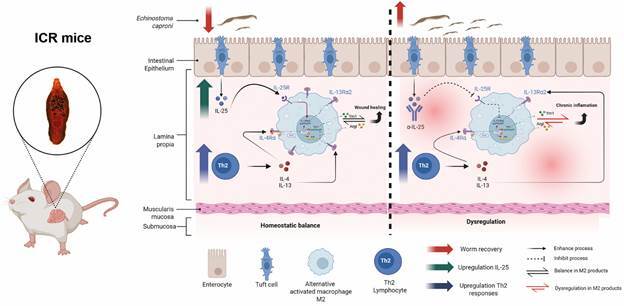
primary infection with *Echinostoma caproni* induces a state of concomitant immunity in Institute of Cancer Research (ICR) mice. Primary infection in mice induces a state of partial resistance or concomitant immunity against homologous superimposed infections by *E. caproni*. This resistance is favoured by an increase in IL-25 expression, alternative activation of macrophages (M2), and the regulation of anti-inflammatory Th2 responses. The binding of IL-25 to its receptor (IL-25R) could be crucial in regulating the expression of IL-13Rα2 and Ym1. Due to Ym1 expression being mediated by Signal transducer and activator of transcription 6 (STAT6) and induced by IL-4/IL-13, IL-25-induced overexpression of the IL-13Rα2 receptor may be crucial. IL-13Rα2 can sequester IL-13 produced in response to a challenge infection, thereby limiting IL-13-mediated Ym-1 production (left). In contrast, blocking IL-25 using IL-25-neutralising antibodies (α-IL-25) prevents adequate immunoregulatory signalling during homologous superimposed infections with *E. caproni*. In the absence of IL-25, host cells express elevated levels of Ym1 to the detriment of the IL-13 receptor IL-13Rα2, leading to exacerbated Th2-type responses (right). In this context, the recovery of adult worms decreases in the presence of an IL-25-regulated response (homeostasis), whereas it increases in the absence of IL-25. Therefore, the activation of these mechanisms could be fundamental to explaining the resistance observed during homologous superimposed infections with *E. caproni*.

Blocking of IL-25 is essential to explain the upregulation of Ym1 and the subsequent susceptibility to superimposed infection. It has been confirmed that Ym1 expression is mediated by STAT6 and induced by IL-4/IL-13, though IL-13 offers a more powerful in vivo inducement of Ym1.^34^ ^,^ ^35^ ^,^ ^36^ ^,^ ^37^ Thus, the downregulation of the “decoy” receptor IL-13Ra2 induced by IL-25 may be critical. IL-4 and IL-13 share a common receptor known as IL-4Rα chain, but IL-13 also uses IL-13Ra1 for signalling via JAK1 and JAK2 of the Janus kinase (JAK) family. IL-13 binds IL-13Ra1, which complexes with IL-4Rα to form the type-1 signal receptor. However, IL-13 also binds to cell surface and soluble forms of the monomeric type-2 receptor for IL-13 (IL-13Ra2). This receptor lacks of signal transduction machinery acting as a non-signalling decoy receptor, since it limits the availability of IL-13 for binding to the type-1 signalling receptor.^38^ ^,^ ^39^ ^,^ ^40^ ^,^ ^41^ The relevance of IL-13Ra2 in the regulation of protective responses against *E. caproni* infections was previously demonstrated^12^ and our study shows that this protective role may be linked to the ability of IL-25 to enhance IL-13Ra2 production, which ensure pro-wound healing mechanisms and resistance to infection. IL-13Ra2 could “sequester” the produced IL-13 in response to challenge infection and, as a consequence, limit the production of Ym-1 mediated by IL-13. Blocking of IL-25 in superimposed infections strongly reduced the IL-13Ra2 expression without changes in the IL-13 production. Under these conditions, superimposed infections occurred in a milieu with elevated levels of free IL-13 that may bind type-1 signalling receptors and, consequently, increasing the expression of Ym1 and the Ym1-mediated inflammation. Impairment of the IL-25-mediated maintenance of intestinal homeostasis abrogated the resistance to infection (Fig. 8). In fact, the mechanisms through which IL-25 generates resistance to *E. caproni* infections in mice are mediated by the activation of tissue regeneration mechanisms and maintenance of intestinal epithelial homeostasis in response to infection.^12^ ^,^ ^13^ ^,^ ^25^ ^,^ ^28^ ^,^ ^29^ In this context, further studies on the role of IL-13, IL-25 and Ym1 can be very useful to obtain a better understanding of the processes associated with resistance to intestinal helminths.

In summary, primary *E. caproni* infections induce concomitant immunity against homologous superimposed infections. This immunity relies on the production of IL-25, rather than in the development of Th2 responses. Despite further studies are required, one of the IL-25-dependent mechanism responsible for immunity appears to be related to the ability of IL-25 to induce IL-13Ra2 upregulation which serves to limit the production of other regulatory proteins such as Ym1 that may interfere with the maintenance of intestinal epithelial architecture.
