# Supplementary material for: Concomitant immunity against superimposed homologous Echinostoma caproni infections in mice is mediated by interleuquin-25
Source: Mem Inst Oswaldo Cruz. 2025 Oct 20;120:e250004. doi: 10.1590/0074-02760250004 (PMC12543364; doi:10.1590/0074-02760250004)
Supplement: Supplementary file 1 [file 1678-8060-mioc-120-e250004-s.pdf]

TABLE

Applied Biosystems Inventoried assays used

	Assay ID details
β-actin	Mm01205647_g1
IL-4	Mm00445259_m1
IL-12p35	Mn00434165_m1
IL-12p40	Mn00434174_m1
IL-13	Mm99999190_m1
IL-25	Mm00499822_m1
IFN-γ	Mm99999071_m1
IL-13Ra2	Mm00515166_m1
Arg I	Mm00475988_m1
Arg II	Mm00477592_m1
Ym-I	Mm00657889_mH
iNOS	Mm01309897_m1
